# Influenza Vaccinations for All Pregnant Women? Better Evidence Is Needed

**DOI:** 10.3390/ijerph15092034

**Published:** 2018-09-18

**Authors:** Alberto Donzelli

**Affiliations:** Consiglio direttivo Fondazione Allineare Sanità e Salute, 20122 Milano, Italy; adonzelli1@libero.it or adonzelli@ats-milano.it

**Keywords:** influenza vaccination, influenza-like illness, pregnant women, stage of pregnancy, first trimester-healthy vaccinee bias, Cochrane Systematic Review, number needed to vaccinate, inflammatory effects

## Abstract

Pregnant women are a World Health Organization (WHO) priority group for influenza vaccination, but evidence of effectiveness and safety for pregnant women comes from observational studies, which are notoriously prone to confounding by indication and healthy-vaccinee bias. The latter type of bias leads to an overestimation of the effectiveness and safety of the vaccine, which may be what occurs in pregnant women. Indeed, better educated women with healthier behaviors and who seek better medical care may be more adherent to vaccinations recommended by doctors, scientific societies and health authorities. Therefore, it is fundamental to obtain information about vaccine effectiveness and safety from randomized controlled trials (RCTs). Cochrane reviews have identified only one RCT with “low risk of bias”. Its results were unclear in terms of maternal, perinatal, and infant deaths and hospitalization, and showed a Number Needed to Vaccine (NNV) of 55 for mothers, with an excess of local adverse effects. A Cochrane review concluded that the inactivated influenza vaccine provides pregnant women with uncertain or very limited protection against influenza-like illnesses and influenza. Some observational studies have suggested possible adverse effects of the inflammation following the vaccination. Consistent with the Cochrane reviewers’ conclusions, further trials for influenza vaccines with appropriate study designs and comparison groups are required before promoting universal seasonal influenza vaccinations of pregnant women. Meanwhile, vaccination in second to third trimester should be offered while communicating the uncertainties that still exist, promoting informed choices. Vaccination in the first trimester is debatable and debated. This does not mean leaving women defenseless; many other useful behavioral and environmental measures can reduce infectious disease.

## 1. Introduction

The World Health Organization [1,2,3] considers pregnant women as a priority group for seasonal influenza vaccination due to their greater susceptibility to severe influenza from the second trimester to the postpartum period. The WHO still recommends annual inactivated influenza virus vaccines for pregnant women, regardless of their stage of pregnancy [3].

The Italian National Vaccination Prevention Plan recommends seasonal vaccination for women who are in the second and third trimesters of pregnancy at the beginning of the epidemic season [4]. There is awareness that the efficacy of the flu vaccine varies in any given year, but the real effectiveness can only be calculated a posteriori.

However, the available evidence comes almost exclusively from observational studies. A systematic review of fetal death, spontaneous abortion, and congenital malformation safety outcomes associated with influenza vaccination during pregnancy [5] cited a recently published randomized controlled trial (RCT) [6], although it added that it was not included in the review.

This work aims to critically re-discuss evidence with respect to the universal influenza vaccination of pregnant women, and requests better evidence about effectiveness and safety.

### 1.1. Evidence from Observational Studies

Unfortunately, observational studies are prone to bias, in particular to confounding by indication and healthy vaccinee bias. The former is more obvious, and occurs as patients with underlying chronic conditions are more likely to be vaccinated than healthy study participants, leading to an underestimation of vaccine effectiveness since the less healthy population is at higher risk of adverse health outcomes. The second bias refers to an alternative situation, where patients in poorer health condition (with functional impairment or other less codified comorbidities, or those closer to death) are less likely to adhere to the annually recommended influenza vaccination. Subjects who adhere to preventive therapies are at the same time more likely to engage in behaviors consistent with a healthy lifestyle than are patients who do not adhere to such treatments [7,8]. Aspects of a healthy lifestyle could include diet, exercise, moderation of alcohol intake, avoidance of illegal drugs or risky behaviors, and seeking better quality health assistance. These characteristics, which are unmeasured in typical pharmaco-epidemiologic databases, may be associated with morbidity and mortality outcomes in observational studies.

This second type of bias leads to an overestimation of vaccine effectiveness (and safety).

To test whether residual confounding by healthy vaccinee effects is still present in the adjusted data, some authors proposed obtaining estimates for time periods outside influenza seasons.

An attempt to systematically investigate these two forms of confounding/bias [9] showed that statistical adjustment for confounders corrected the confounding by indication bias, at least partially. However, despite adjustment, many studies still evidenced significant estimates of implausible vaccine effectiveness outside the influenza season, indicating unmeasured confounding due to healthy vaccinee bias.

In the setting of pregnant women, this bias may be strongly operating, because more health-conscious and better educated women with overall healthier behaviors and who seek better medical care may be more adherent to influenza vaccination, which is highly recommended by doctors, scientific societies, and health authorities [10]. It was also shown that pregnant women who receive a recommendation for the influenza vaccine from their obstetrician or obstetric provider are 5- to 50-fold more likely to be immunized [10]. In contrast, pregnant women with a lower education and those of foreign origin are more vulnerable to non-vaccination [11]. Moreover, in England, with each quintile of decrease in socio-economic position, women were shown to be 25% less likely to have had any antenatal care [12].

Therefore, while in the elderly confounding by indication and healthy vaccinee bias are both present and operating in opposite directions, in pregnant women confounding by indication is weaker, if not absent, and the healthy vaccinee bias is stronger.

### 1.2. Evidence from Randomized Controlled Trials

For the above reasons, it is fundamental to consider information on the effectiveness and safety of vaccine coming from existing randomized controlled trials (RCTs). A Cochrane Systematic Review [13,14] that aimed to assess the impact of influenza vaccination during pregnancy on maternal, neonatal, and infant health outcomes as compared to placebo/control has found only one RCT of high validity, conducted in Soweto, South Africa (upper-middle income country), including 2116 women with estimated gestation of 20 to 24 weeks and 2049 neonates. The RCT was judged to be at “low risk of bias”.

Despite inactivated influenza vaccination (IIV3) during pregnancy being associated with a decrease in confirmed influenza among women and their babies (vaccine efficacy rates of 50.4% and 48.8%, respectively), the impact of clinical outcomes was somehow disappointing. The authors found that there was no clear difference between the influenza vaccine and placebo control group in most of the review’s primary outcomes: there were two maternal deaths, both in the vaccine group (RR 4.96, 95% CI 0.24 to 103.24, moderate quality evidence). The numbers of perinatal deaths (stillbirths and deaths in the first week of life) were 15 and 12 in the vaccine group, and 9 and 10 in the placebo group, respectively (RR 1.32, 95% CI 0.73 to 2.38, moderate quality evidence). The numbers of infant deaths up to 175 days after birth were 15 in the vaccine group, and 21 in the placebo group (RR 0.71, 95% CI 0.37 to 1.37, moderate quality evidence). Although the sum of deaths in the two groups was nearly the same, some differences in adverse events, while not significant, show tendencies that are not reassuring (Table 1).

One could speculate that the modest/moderate inflammatory stress linked to vaccination [15,16,17] might not be so benign for some predisposed and frail fetuses, encouraging unfavorable outcomes to some extent.

Moreover, the data showed no differences in terms of influenza-like illness in women (RR 0.96, 95% CI 0.79 to 1.16) or their babies (RR 1.02, 95% CI 0.94 to 1.09), or any respiratory illness in women (RR 0.97, 95% CI 0.91 to 1.04, high quality evidence) or their babies (RR 1.01, 95% CI 0.95 to 1.07, high quality evidence). The review [13] did not find significant differences between the two groups in terms of maternal hospitalization for any infection (RR 2.27, 95% CI 0.94 to 5.49; moderate quality evidence), and for neonatal hospitalization for sepsis (RR 1.60, 95% CI 0.73 to 3.50; moderate quality evidence). It can be noted that, while the differences for all these outcomes did not reach statistical significance, their tendencies were often not in the expected and desired direction.

For local and systemic reactions, the RCT [6] declared that “Injection-site reactions (mainly mild to moderate) were more frequent among IIV3 recipients than among placebo recipients in both cohorts, but there were no other significant differences in solicited reactions between the two study groups in either cohort”. This claim is debatable, for various reasons.

First, the majority of local reactions (tenderness, erythema, swelling, induration, bruising) would have only minimal or no chance of occurring in situ in the absence of a placebo injection. Therefore, for this type of reaction, the correct comparison should not be made with what occurred with a placebo injection, but with doing nothing. Furthermore, in the context of a double-blind RCT, the patient and the study personnel are unaware of whether the injection contains an active drug, and therefore a nocebo effect could be added to the effect of injection of a saline solution.

Second, not all the local reactions were mild or moderate: some were defined as severe (in the IIV3 recipients: tenderness 3.9%, induration 1.7%, bruising 1.7%, and at least one severe reaction 5%), and, as specified above, a proper control group should have “no reaction in the point of no injection”.

Third, for systemic reactions for which a control group has a natural background rate, particularly weakness/tiredness, fever, and joint pain, the vaccinated group showed a (plausible) tendency to more severe reactions. Moreover, also in the case of systemic reactions a blind control group receiving a saline injection might not be exempt from a potential nocebo effect.

The authors conclude that further trials are needed for viral influenza vaccines with appropriate study designs and suitable comparison groups.

A recent update of a Cochrane Review on vaccines for preventing influenza in healthy adults [14] again included only the aforementioned RCT [6] (at low risk of bias) and one controlled clinical trial (at high risk of bias), assessing the effects of vaccination in pregnant women. The efficacy of inactivated vaccine containing pH1N1 against influenza was 50% (95% CI 14% to 71%) in mothers (NNV 55), and 49% (95% CI 12% to 70%) in infants up to 24 weeks (NNV 56). No data were available on efficacy against seasonal influenza during pregnancy. Evidence from observational studies, more subject to bias, showed the effectiveness of influenza vaccines against influenza-like illness (ILI) in pregnant women to be 24% (95% CI 11% to 36%, NNV 94), and against influenza in infants from vaccinated women to be 41% (95% CI 6% to 63%, NNV 27).

The authors [14] conclude that protection against influenza and ILI in mothers and newborns was smaller than the effects seen in other populations considered in their review. They state that the protection provided to pregnant women against ILI and influenza by the inactivated influenza vaccine is uncertain, or at most this was minimal. These conclusions should not be dismissed relying only on the results of observational studies, subject to well-known confounding.

### 1.3. Possible Adverse Effects Cannot Be Categorically Excluded

In high-income countries with a relatively low number of severe cases and deaths of influence in pregnancy (e.g., in Italy in the last five annual influenza seasons there were 31 severe cases overall in pregnant women, of which 3 resulted in deaths), influenza vaccination in the first trimester should not be encouraged. This is in view of potential neurodevelopmental risks shown in some studies [16,17]. Other studies adding some evidence to the possible pathogenetic pathway mediated by inflammation [15,18,19]. Indeed, investigating the association between influenza infection and vaccination during pregnancy and autism spectrum disorder (ASD) risk in a cohort study of 196,929 children born at Kaiser Permanente Northern California, Zerbo and colleagues [16] found that influenza vaccination anytime during pregnancy was not associated with ASD risk (the adjusted HR was of borderline significance: 1.10; 95% CI 1.00–1.21). A suggestion of increased ASD risk among children whose mothers received an influenza vaccination in their first trimester (adjusted HR 1.20; 1.04–1.39) “was not statistically significant after adjusting for multiple comparisons” [16]. This conclusion was doubted, [17] because Zerbo and colleagues used the stringent Bonferroni’s correction, which only adjusts for independent comparisons that they arbitrarily elevated to eight.

Instead, it is biologically plausible that eventual effects on the fetal nervous system occur in the first trimester, during embryogenesis, and therefore it would have been logical to perform the main analysis in the first trimester. In their reply, Zerbo and colleagues [20] agree that eight independent comparisons were not justified, but again arbitrarily reduced them to six. They could legitimately have reduced the independent comparisons to three, one for each trimester of pregnancy: this logical correction would have maintained the statistical significance of the excess of ASD risk in the first trimester, even on adopting the Bonferroni adjustment which is more conservative than other equally legitimate methods of correction for multiple testing.

The main problem, however, is that influenza vaccination induces an inflammatory response during pregnancy [15,18,19]. Magnitude and duration of this inflammatory response is lower and shorter than that induced by the viral infection. However, there is considerable variability in magnitude, up to a change from baseline to 991% and 728% for IL-6, respectively one and two days post-vaccination; and changes in CRP and TNF-α up to 1126% and 1521% respectively, one week post-vaccination [15]. However, the rates of inflammatory responses from vaccination and from influenza illness are very different. The Cochrane review [14] demonstrated that, for every 100 healthy adults vaccinated, only 1.4 to 3.4 influenza or ILI cases are prevented: NNV 29 to 71. For pregnant women the NNV was 55.

Therefore, at a population level, the inflammatory effects of 100 vaccinated women should be compared with those caused in about two women with influenza illness. In addition, the data reported in [16] (Table 3) show that the inflammatory effect of influenza infection during the first trimester of pregnancy involved only 2/1000 women (443/196,929), while the vaccination effect was found in over 297/1000 vaccinated women (13,477/45,231).

Another study [21] found a significant association of spontaneous abortion with receipt of inactivated influenza vaccine in 2010–2011 and 2011–2012, and reported other studies showing potential relationships between vaccination and inflammation, and inflammation and pregnancy loss.

## 2. Conclusions

In my opinion, consistent with the conclusions of the Cochrane reviewers [13,14], further RCTs for viral influenza vaccines with appropriate study designs are required before promoting universal seasonal influenza vaccination of pregnant women, for which current evidence is insufficient.

These RCTs should be large and pragmatic, with suitable comparison groups (including one in which the intervention is “to do nothing”) and long-term follow-up. Only these trials can avoid the possible flawed picture of observational studies where many of the benefits are only hypothesized, and possible serious harms are underestimated, both for the healthy-vaccinees bias and for the known tendency of passive pharmacovigilance to underreporting of adverse events, even serious ones, but with a fairly high background rate.

To overcome a typical ethical objection (“it would be unethical to deny the benefits of the vaccine to the control group”), these trials should include only women persistently hesitant about vaccination, despite balanced and comprehensive information on the existing evidence and the areas of uncertainty about potential benefits and risks.

In the meantime, health services could offer the vaccination in the second and third trimester, but without hiding the uncertainties still existing, and promoting balanced information and an informed choice.

Moreover, it seems reasonable to apply the precautionary principle and refrain from vaccinating in the first trimester of pregnancy, unless an informed woman requests it.

The above proposal especially refers to high-income countries, where the harms of influenza to pregnant women and to their fetuses might be less dramatic, and the probabilities of influenza infection for a pregnant woman are lower in terms of the demographic structure of the population and of families.

### Other Recommendations to Limit Respiratory Infections

Reducing the urge for universal influenza vaccination of pregnant women does not mean leaving them without defenses, because many other useful measures can reduce the chance of getting sick. The United States Center for Disease Control (US CDC) [22] recommends: washing hands often with water and soap (if not available, with an alcohol-based hand rub); avoiding touching eyes, nose, and mouth; staying away from sick people; making a plan for others to care for sick people suspected to have influenza in the household; and encouraging cough etiquette and hand hygiene among all close contacts.

Other useful measures can be:

- Not smoking and avoiding smoky environments. Smokers have more respiratory infections, illnesses, and complications [23].
- If someone in the house is sick, avoid antipyretics to treat fever as much as possible: they might increase and extend the transmission of associated infections. [24] At the population level, the data suggest that fever suppression increases the expected number of influenza cases and deaths in the United States, with an estimated increase of 5% (95% CI: 0.2–12.1%) [25].
- Practicing other healthy habits: Avoid overwork and ensure rest, because stress lowers defenses. Eat and drink healthy foods, and exercise regularly (while avoiding over-exercise) to maintain optimal immune function
- Avoiding closed and crowded places in the weeks in which the influenza epidemic is locally occurring, preferring low-traffic hours or/and wearing masks (OR 0.32, 95% CI 0.25 to 0.40; Number Needed to Treat (NNT) = 6, 4.54 to 8.03) [26].

## Figures and Tables

**Table 1:** Adverse events in the arms of vaccinated and unvaccinated mothers [6,13].

Outcomes	Vaccinated	Unvaccinated	RR, 95% CI
Maternal deaths	2	-	4.96, 95% CI 0.24–103.24
Stillbirth	15	9	1.32, 95% CI 0.73–2.38
Death in the first week of life	12	10	
Infant death up to 175 days after birth	15	21	0.71, 95% CI 0.37–1.37
Local and systemic reactions	Clear disadvantage for the vaccinated women		
NNV (to avoid 1 influenza)	55 and 56 (for mothers and for infants, respectively) [14]		
